# Pathophysiology and clinical implications of the veno-arterial PCO_2_ gap

**DOI:** 10.1186/s13054-021-03671-w

**Published:** 2021-08-31

**Authors:** Zied Ltaief, Antoine Guillaume Schneider, Lucas Liaudet

**Affiliations:** 1 Service of Adult Intensive Care Medicine, Lausanne University Hospital, 1011 Lausanne, Switzerland; 2 Unit of Pathophysiology, Faculty of Biology and Medicine, University of Lausanne, 1011 Lausanne, Switzerland

## Abstract

This article is one of ten reviews selected from the Annual Update in Intensive Care and Emergency Medicine 2021. Other selected articles can be found online at https://www.biomedcentral.com/collections/annualupdate2021. Further information about the Annual Update in Intensive Care and Emergency Medicine is available from https://link.springer.com/bookseries/8901.

## Introduction

The persisting high mortality of circulatory shock highlights the need to search for sensitive early biomarkers to assess tissue perfusion and cellular oxygenation, which could provide important prognostic information and help guide resuscitation efforts. Although blood lactate and venous oxygen saturation (SvO_2_) are commonly used in this perspective, their usefulness remains hampered by several limitations. The veno-arterial difference in the partial pressure of carbon dioxide (Pv-aCO_2_ gap) has been increasingly recognized as a reliable tool to evaluate tissue perfusion and as a marker of poor outcome during circulatory shock, and it should therefore be part of an integrated clinical evaluation. In this chapter, we present the physiological and pathophysiological determinants of the Pv-aCO_2_ gap and review its implications in the clinical assessment of circulatory shock.

## Physiological aspects of CO_2_ production and transport

**Fig. 1:**
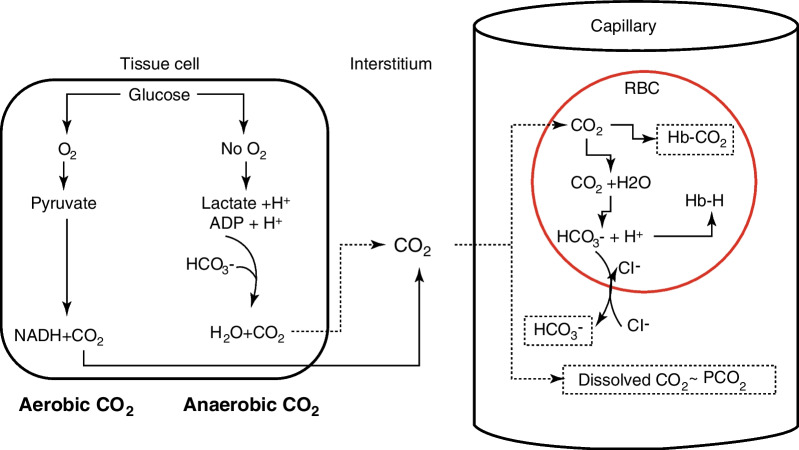
Physiology of CO_2_ production and transport. In cells, CO_2_ is produced (in mitochondria) as a byproduct of substrate oxidation. Under anaerobic conditions, CO_2_ is generated in small amounts, as the results of HCO_3_^−^ buffering of protons released by lactic acid and the hydrolysis of ATP. CO_2_ diffuses into the interstitial tissues and then into capillaries, where it is transported as dissolved CO_2_ in plasma (in equilibrium with the PCO_2_), bound to hemoglobin as carbamino-hemoglobin (HbCO_2_) in red blood cells (RBC), and as HCO_3_^−^, following the reaction of CO_2_ with H_2_O within RBC, a reaction catalyzed by carbonic anhydrase to form HCO_3_^−^ and H^+^. HCO_3_^−^ exits the RBC in exchange with chloride anions (Cl^−^), whereas protons are buffered by hemoglobin, forming HbH

Under aerobic conditions, CO_2_ is produced at the mitochondrial level as a by-product of substrate oxidation (pyruvate and citric acid cycle intermediates) (Fig. 1). The relationship between the amount of oxygen consumed (VO_2_) and CO_2_ produced (VCO_2_) during aerobic metabolism is termed the respiratory quotient (RQ = VCO_2_/VO_2_), and differs according to the main type of oxidized substrate (glucose, RQ = 1; proteins, RQ = 0.8; lipids, RQ = 0.7). Under anaerobic conditions, protons (H^+^) resulting from lactic acid production and ATP hydrolysis may generate CO_2_ following buffering by bicarbonates (HCO_3_^−^), leading to the formation of so-called “anaerobic CO_2_” [1]. Once formed, CO_2_ diffuses within the surrounding environment and capillary blood, to be transported to the lungs for elimination. In blood, CO_2_ transport is partitioned into three distinct fractions [2]:

1. Dissolved CO_2_ fraction, which is in equilibrium with the partial pressure of CO_2_ (PCO_2_), according to Henry’s law of gas solubility: V_gas_ = S_gas_ × (P_gas_/P_atm_), where V_gas_ is the volume of dissolved gas (in ml/ml), S_gas_ is the Henry’s constant of gas solubility (0.52 ml/ml for CO_2_ at 37 °C), and P_atm_ the atmospheric pressure. Thus, in arterial blood with a PaCO_2_ of 40 mmHg (at sea level, 37 °C), dissolved CO_2_ = [0.52 × (40/760)] = 27 ml/l, which is about 5% of the total CO_2_ (note that, in mmol/l, Henry’s constant for CO_2_ = 0.03 mmol/l/mmHg; also note that the conversion factor from mmol to ml CO_2_ is ~ 22.3).
2. Bicarbonate (HCO_3_^−^). CO_2_ in blood readily diffuses within red blood cells (RBCs), where it combines with H_2_O to form carbonic acid (H_2_CO_3_), a reaction catalyzed by the enzyme carbonic anhydrase. In turn, H_2_CO_3_ dissociates to form HCO_3_^−^ and H^+^. While H^+^ is buffered by hemoglobin (formation of HbH), HCO_3_^−^exits the RBC in exchange for a chloride anion (Cl^−^) via a HCO_3_^−^-Cl^−^ transporter (erythrocyte chloride shift or Hamburger effect). Thus, the HCO_3_^−^ concentration increases in venous blood whereas the Cl^−^ concentration diminishes. CO_2_ transport as HCO_3_^−^ (RBC and plasma fraction) represents about 90% of the total CO_2_ content in arterial blood (this proportion is lower in venous blood due to the Haldane effect). Taking into account a normal hematocrit of 0.45, the CO_2_ content under the form of HCO_3_^−^ (in whole blood) is ~ 435 ml/l.
3. Formation of carbamino compounds within hemoglobin: part of the CO_2_ within the RBC combines with free amino (R-NH_2_) groups within hemoglobin to form carbamino-hemoglobin (R-NH_2_-CO_2_). This reaction is enhanced when hemoglobin carries less oxygen, implying that more CO_2_ is transported as (R-NH_2_-CO_2_) when the PO_2_ decreases, which is the basis of the Haldane effect described below. CO_2_ transport under the form of (R-NH_2_-CO_2_) represents about 5% of the total CO_2_ content in arterial blood (~ 1.1 mmol/L ≈ 25 ml/l).

In summary, the total CO_2_ content of blood under physiological conditions equals:

$$[{\text{Dissolved}}\,{\text{CO}}_{2} ] + \left[ {{\text{HCO}}_{3}^{ - } } \right] + \left[ {{\text{R}} - {\text{NH}}_{2} - {\text{CO}}_{2} } \right]$$

which is ≈ 490 ml/l in arterial blood and ≈ 535 ml/l in mixed venous blood, hence a veno-arterial difference of approximately 45 ml/l. A more precise calculation of the CO_2_ content of blood can obtained by the Douglas equation, but this is too complex to be calculated at the bedside [3].

### The CO_2_ dissociation curve (PCO_2_-CCO_2_ relationship)

**Fig. 2:**
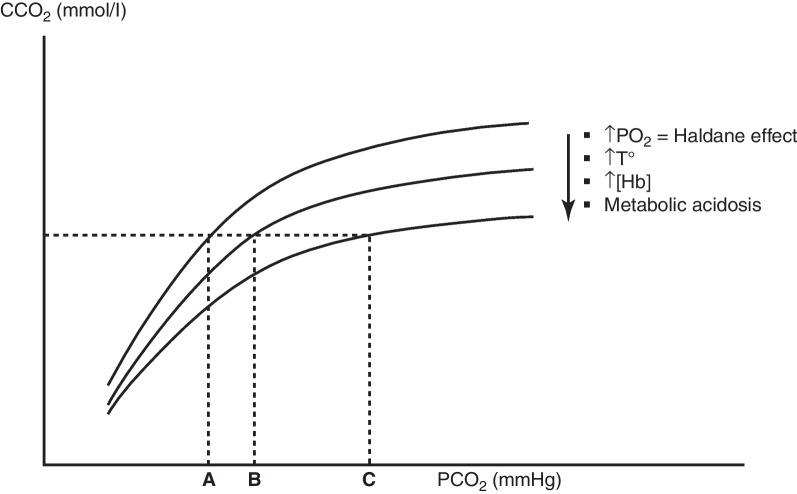
The CO_2_ dissociation curve. A curvilinear relationship exists between CO_2_ partial pressure (PCO_2_) and CO_2_ content (CCO_2_), so that PCO_2_ = *k* × CCO_2_. At low values of PCO_2_, the slope of the relationship is steeper, implying a smaller increase of PCO_2_ at any CCO_2_ than at high values of PCO_2_, where the slope of the relationship flattens. The position of the relationship is modified by various factors. A rightward and downward shift of the curve, corresponding to an increase of the k coefficient is produced by high PaO_2_ (Haldane effect), elevated temperatures, high hemoglobin concentrations and metabolic acidosis. A rightward shift of the curves implies that, for a same CCO_2_, the PCO_2_ increases, as indicated by the points A, B and C

As is the case for oxygen, a relationship exists between the PCO_2_ and the CO_2_ content (CCO_2_) of blood (Fig. 2). However, in contrast to the sigmoid shape of the O_2_ dissociation curve, the CO_2_ dissociation curve is slightly curvilinear, indicating a proportional increase in CCO_2_ over a wide range of PCO_2_. In the physiological range, the relationship between CCO_2_ and PCO_2_ can therefore be resolved by the equation:

1 $${\text{PCO}}_{2} = k \times {\text{CCO}}_{2}$$

Important information provided by the PCO_2_-CCO_2_ relationship is the shift produced at different values of oxygen saturation of hemoglobin (HbO_2_). Indeed, as hemoglobin gets saturated with O_2_, it can carry less CO_2_ as carbaminoHb, and inversely. This behavior is known as the Haldane effect, which implies that for a same PCO_2_, CCO_2_ is higher at lower HbO_2_ saturation. In other words, this means that as the k constant in the relationship above decreases, the PCO_2_-CCO_2_ curve is shifted to the left. The consequence of this effect is that, in tissues, more CO_2_ is loaded by Hb as it releases O_2_, allowing PCO_2_ to increase only moderately (from 40 to 46 mmHg), in spite of a marked increase in CCO_2_ due to the tissue production of CO_2_. Without the Haldane effect, the venous PCO_2_ would increase significantly more for a similar increase in CO_2_ content.

The curvilinearity of the CO_2_ dissociation curve indicates that CCO_2_ increases more steeply at low values of PCO_2_ and is more flat at high PCO_2_ values. It is also noticeable that the curve can be displaced by a certain number of factors: In conditions of metabolic acidosis, the reduction in HCO_3_^−^ due to H^+^ buffering reduces the formation of carbamino (R-NH_2_-CO_2_) compounds inside hemoglobin [4]. As a result, for a given CCO_2_, the PCO_2_ must increase, which means an increase in the *k* constant, and a rightward shit of the relationship. The opposite occurs under conditions of metabolic alkalosis. Other factors influencing the curve are the hematocrit and temperature. At increasing hematocrit, there is a decrease in plasma space with a reduction of HCO_3_^−^ and a decrease in CO_2_ content at any value of PCO_2_, with a shift to the right of the curve. At increasing temperatures, the reduced CO_2_ solubility also shifts the relationship to the right [4]. These considerations imply, therefore, that PvCO_2_ may vary at constant total venous CCO_2_ according to the particular conditions (HbO_2_ saturation [i.e., the Haldane effect], arterial pH, temperature and hematocrit).

## The Pv-aCO_2_ gap: pathophysiology and clinical implications

A discussed earlier, the CCO_2_ in the venous side of the circulation is determined by the aerobic production of CO_2_ in tissues, influenced by the metabolic rate and the respiratory quotient, and may also increase via non-aerobic production of CO_2_. The generation of CO_2_ de facto increases the CCO_2_ on the venous side of the circulation, implying an obligatory difference between arterial and venous CCO_2_, termed the veno-arterial difference in CCO_2_, or veno-arterial CCO_2_ gap: va-CCO_2_ gap = (venous - arterial) CCO_2_ [1].

The tissue VCO_2_ does not accumulate under normal conditions, being washed out by the blood flowing across the tissue and eliminated by the lungs. Accordingly, any reduction in tissue blood flow (stagnant condition) will result in an accumulation of tissue CO_2_, implying an increase in the va-CCO_2_ gap, in accordance with Fick’s principle:

$${\text{VCO}}_{{2{\text{tissue}}}} = \left[ {\left( {{\text{Blood}}\,{\text{flow}}_{{{\text{tissue}}}} \times \left( {{\text{va}} - {\text{CCO}}_{2} \,{\text{gap}}_{{{\text{tissue}}}} } \right)} \right)} \right]$$

At the systemic level, the relationship is:

$${\text{VCO}}_{2} = \left[ {\left( {{\text{Cardiac}}\,{\text{output}} \times \left( {{\text{va}} - {\text{CCO}}_{2} \,{\text{gap}}} \right)} \right)} \right]$$

According to the equation (PCO_2_ = *k* × CCO_2_), the Fick equation for CO_2_ can be rewritten as:

$$k \times {\text{VCO}}_{2} = \left[ {{\text{Cardiac}}\,{\text{output}} \times \left( {{\text{Pv}} - {\text{PaCO}}_{2} } \right)} \right]$$

and

$$\left( {{\text{Pv}} - {\text{PaCO}}_{2} } \right) = \left[ {\left( {k \times {\text{VCO}}_{{2}} } \right)/{\text{Cardiac}}\,{\text{output}}} \right]$$

Therefore, the Pv-aCO_2_ gap represents a very good surrogate indicator of the adequacy of cardiac output and tissue perfusion under a given condition of CO_2_ production. The normal Pv-aCO_2_ gap is comprised between 2 and 6 mmHg [5], and many studies assessing Pv-aCO_2_ gap in clinical conditions used a cut-off value of 6 mmHg above which the gap is considered abnormally elevated. Although the venous PCO_2_ should ideally be obtained in a mixed venous blood sampling, good agreement between central and mixed venous PCO_2_ values has been reported [6]. Therefore, both central and mixed venous PCO_2_ can be used for the calculation of the va-CO_2_ gap, as long as the variables are not interchanged during treatment in a given patient.

### The inverse relationship between cardiac output and the Pv-aCO_2_ gap

**Fig. 3:**
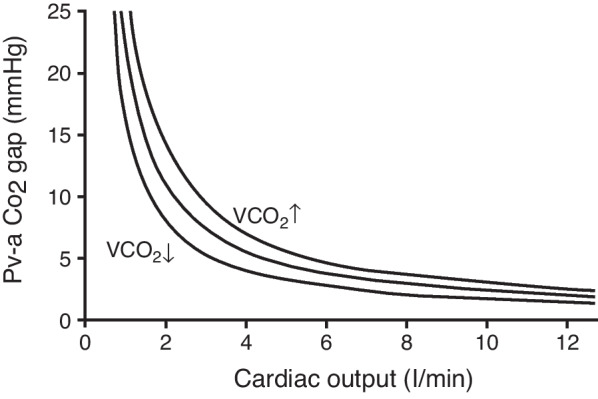
The inverse relationship between cardiac output and the Pva-CO_2_ gap. A reduction in cardiac output is associated with a progressive increase in the Pva-CO_2_ gap, which becomes exponential at very low cardiac output values, because of the flat slope of the CO_2_ dissociation curve in conditions of tissue hypercarbia. The relationship is displaced to the right at higher CO_2_ production (VCO_2_)

The inverse relationship between cardiac output and the Pv-aCO_2_ gap (Fig. 3) has been repeatedly demonstrated in both experimental [7] and clinical [8] settings. It is noteworthy that this relationship is not linear, but curvilinear (Fig. 3). At very low cardiac output, the (Pv-aCO_2_ gap) indeed increases more rapidly. This large increase in Pv-aCO_2_ gap is primarily due to the flattened relation between CCO_2_ and PCO_2_ at high values of CCO_2_ in conditions of tissue hypercarbia [5], and this is further magnified if tissue metabolic acidosis develops, due to the rightward shift of the PCO_2_-CCO_2_ relationship in acidic conditions (increased *k* coefficient, see above). Also, venous accumulation of CO_2_ will increase as a consequence of low pulmonary perfusion and CO_2_ elimination, further widening the gap [9]. In contrast, the increase in Pv-aCO_2_ in very low flow states with conditions of VO_2_-oxygen delivery (DO_2_) dependence will be attenuated by the mandatory reduction in aerobic VCO_2_. Such a decrease in VCO_2_ results in a leftward shift of the cardiac output/Pv-aCO_2_ gap relationship, as shown in Fig. 3 [5].

### Pv-aCO_2_ gap and tissue dysoxia

In addition to tracking changes in cardiac output and tissue perfusion, the Pv-aCO_2_ gap can increase through an augmentation of VCO_2_ [8]. Under *aerobic* conditions, that is in the absence of any clinical sign of shock or increased blood lactate, such an increase reflects an increased metabolic demand or an increase in RQ (glucidic diet), or both. Physiologically, an increased metabolic rate is generally coupled with an increase in cardiac output, but such adaptation may not occur in critically ill patients with inadequate cardiovascular reserves, which may result in an increased Pv-aCO_2_ gap. Interventions should here be targeted first to reduce the metabolic demand. Persistence of an increased Pv-aCO_2_ gap should not necessarily prompt therapies to increase cardiac output, given the risk associated with deliberate increase in cardiac output in the absence of tissue dysoxia [10]. However, it is noteworthy that an increased Pv-aCO_2_ gap immediately after surgery in high risk patients, independent of their hemodynamic condition, SvO_2_ and lactate, has been associated with significantly more complications [11]. This suggests that a high Pv-aCO_2_ gap could track insufficient resuscitation and might represent a goal for hemodynamic optimization in such patients, but this issue is controversial and remains to be proven [9].

Under *anaerobic* conditions, the question as to whether the Pv-aCO_2_ gap can be used as a marker of tissue dysoxia, by detecting increased anaerobic VCO_2_ from H^+^ buffering, has attracted much attention. An advantage of Pv-aCO_2_ gap in this sense would be its ability to rapidly track changes in CO_2_ formation, hence providing sensitive, rapid and continuous detection of ongoing anaerobiosis. This would contrast from usual markers of tissue dysoxia, such as SvO_2_ or lactate. Indeed, SvO_2_ can be unreliable in conditions of reduced oxygen extraction and hyperdynamic circulation (sepsis) [12]. The disadvantage of lactate is its lack of specificity as a marker of dysoxia (type A vs type B hyperlactatemia), and its relatively slow clearance kinetics dependent on liver perfusion and function [13], which limits its utility to rapidly track changes in tissue oxygenation [9].

#### The Pv-aCO_2_ gap in stagnant dysoxia

In essence, tissue dysoxia is classically attributed to stagnant, hypoxic, anemic and cytopathic mechanisms. As a sensitive marker of reduced cardiac output, an increased Pv-aCO_2_ gap is a reliable indicator of stagnant dysoxia. Importantly, the major gap noted under very low flow conditions (see earlier) has been associated with a global reduction in VCO_2_ (VO_2_-DO_2_ dependence), implying that any increase in anaerobic VCO_2_ could not offset the depressed aerobic VCO_2_ [7]. Therefore, the increased Pv-aCO_2_ gap depends entirely on the stagnant accumulation of tissue CO_2_, but not on increased anaerobic VCO_2_ in low flow conditions [1, 14].

#### The Pv-aCO_2_ gap in hypoxic or anemic dysoxia

To address the role of the Pv-aCO_2_ gap to detect hypoxic dysoxia, Vallet et al. reduced DO_2_ below the critical threshold in an isolated dog hindlimb model, by reducing blood flow or by decreasing PO_2_ [15]. Both conditions similarly reduced VO_2_ and O_2_ extraction, but the Pv-aCO_2_ gap increased exclusively in the ischemic, but not hypoxic condition, implying that stagnant, but not hypoxic dysoxia was the responsible mechanism [15]. Comparable results were obtained by Nevière et al. in the intestinal mucosa of pigs, following the systemic reduction in DO_2_ to similar levels either by reduction of cardiac output or arterial PO_2_ [16]. With respect to anemic dysoxia, similar conclusions were obtained in sheep hemorrhage models, in which no increase in Pv-aCO_2_ gap was detected under conditions of VO_2_/DO_2_ dependency due to reduced hemoglobin concentration [17], unless there was a concomitant reduction in cardiac output [18]. Hence, significant hypoxic or anemic dysoxia occurs in the absence of any Pv-aCO_2_ gap increase.

#### The Pv-aCO_2_ gap in cytopathic dysoxia

An acquired intrinsic abnormality of tissue O_2_ extraction and cellular O_2_ utilization, primarily related to mitochondrial impairment, defines the concept of cytopathic hypoxia, and the resulting cellular bioenergetic failure could represent an important mechanism of organ dysfunction in sepsis [19]. Mitochondrial defects have been demonstrated in several tissues obtained from animals in various models of sepsis, and limited data also exist on altered mitochondrial metabolism in human biopsy samples or circulating blood cells [20]. The detection of cytopathic hypoxia, however, is still not feasible at the bedside, although new techniques such as the measurement of mitochondrial O_2_ tension using protoporphyrin IX-Triplet State Lifetime Technique (PpIX-TSLT) are currently being developed [21]. Furthermore, impaired O_2_ extraction in sepsis does not necessary imply cytopathic hypoxia, as it may be related to impaired microcirculation.

Theoretically, the increased anaerobic CO_2_ generation in conditions of cytopathic hypoxia could result in increased anaerobic VCO_2_ leading to an increased Pv-aCO_2_ gap. This assumption has been evaluated in a porcine model of high dose metformin intoxication, which induces mitochondrial defects comparable to cyanide poisoning [22]. As expected, treated pigs exhibited reduced VO_2_ and marked lactic acidosis, in spite of preserved systemic DO_2_. However, although VCO_2_ decreased less than VO_2_, suggesting some anaerobic VCO_2_, no significant increase in Pv-aCO_2_ gap was noted. In a human case report of massive metformin intoxication, Waldauf et al. also reported no elevation in Pv-aCO_2_ gap despite major lactic acidosis and reduced aerobic VO_2_, as detected by increased SvO_2_ [23]. Therefore, although data are very limited, cytopathic dysoxia related to impaired mitochondrial respiration appears not to widen the Pv-aCO_2_ gap.

### The Pv-aCO_2_ gap in sepsis

Ongoing tissue dysoxia with persistent lactic acidosis is a hallmark of sepsis, and associated with a poor prognosis. Although a hyperdynamic circulation is characteristic of sepsis, many septic patients may have a cardiac output that is insufficient to meet metabolic demands, because of persistent hypovolemia or concomitant myocardial dysfunction. An increased Pv-aCO_2_ gap has been reported in patients with lower cardiac output in sepsis, consistent with the ability of the Pv-aCO_2_ gap to detect stagnant dysoxia, also in the context of sepsis [24]. In such conditions, an increase in cardiac output correlates with a parallel decrease in Pv-aCO_2_ gap [25]. Importantly, as reported by Vallee et al. [26], the Pv-aCO_2_ gap is able to detect persistently low cardiac output even in patients with a normal SvO_2_. Such a high Pv-aCO_2_ gap during the early resuscitation of septic shock has been correlated with more organ dysfunction and worse outcomes [27].

Many septic patients display persistent lactic acidosis in spite of an elevated cardiac output and normal or even increased SvO_2_. This implies that mechanisms unrelated to macrohemodynamics sustain tissue dysoxia in this setting, i.e., a loss of so-called hemodynamic coherence, with significant negative impact on outcome [28]. Impaired microcirculatory perfusion is indeed a prototypical perturbation in experimental [29] and human sepsis [30], which may impair tissue oxygenation. Such microcirculatory derangements result in tissue CO_2_ accumulation, which can be tracked, for example, by sublingual capnometry, as shown by Creteur et al. [31]. Accordingly, in a prospective observational study including 75 patients with septic shock, Ospina-Tascon et al. found a significant correlation between Pv-aCO_2_ gap and microcirculatory alterations. These were independent of systemic hemodynamic status and persisted even after correction for the Haldane effect [32], indicating that the Pv-aCO_2_ gap may be a useful tool to assess impaired microcirculation in sepsis [33]. Furthermore, Creteur et al. reported that increasing cardiac output with dobutamine in patients with impaired microcirculation resulted in a decreased regional PCO_2_ gap (sublingual and gastric mucosal) that was associated with a significant increase in well-perfused capillaries [31].

In summary, an elevated (> 6 mmHg) Pv-aCO_2_ gap in sepsis detects stagnant dysoxia, whether related to a low cardiac output or a derangement in microcirculatory blood flow, and this holds true even in the presence of a normal or elevated SvO_2_. As such, a high Pv-aCO_2_ gap might prompt a trial to improve tissue blood flow by increasing cardiac output [34].

Finally, many septic patients with an elevated cardiac output exhibit a normal Pv-aCO_2_ gap, resulting from elevated CO_2_ washout by increased tissue blood flow. Many of these patients still display signs of ongoing dysoxia with lactic acidosis and organ dysfunction. Whether this pattern reflects cytopathic dysoxia or regional microcirculatory alterations not tracked by Pv-aCO_2_ gap elevation remains to be established.

## Use of the Pv-aCO_2_ gap as a prognostic tool

In sepsis, evidence exists that a Pv-aCO_2_ gap > 6 mmHg, even after normalization of blood lactate, is predictive of poor outcomes [35–37], which has been highlighted in a recent systematic review of 12 observational studies [38]. Whether this holds true for a broader population of critically ill patients with circulatory shock has been questioned in a recent meta-analysis of 21 studies with a total of 2155 patients from medical, surgical and cardiovascular ICUs [37]. Overall, a high Pv-aCO_2_ gap was associated with higher lactate levels, lower cardiac output and central venous oxygen saturation (ScvO_2_), and was significantly correlated with mortality. The latter was however restricted to medical and surgical patients, with no association found for cardiac surgery patients. Since the meta-analysis included only two studies in cardiac surgery, this negative result should be interpreted with caution. Three recent retrospective studies not included in the meta-analysis [39–41] indeed reported a negative impact of high postoperative Pv-aCO_2_ gap on major complications and mortality after cardiac surgery, although with limited diagnostic performance [41].

Future studies are needed to refine the value of the Pv-aCO_2_ gap as a prognostic biomarker in cardiac surgery patients, taking into account the low mortality (3.4%) in this population [42].

## Pitfalls in the interpretation of the Pv-aCO_2_ gap

As already mentioned, several factors may influence the position of the PCO_2_-CCO_2_ relationship by influencing the *k* factor of proportionality between both variables (see Fig. 2), which must be taken into account for a proper interpretation of the Pv-aCO_2_ gap. These include the oxygen saturation of hemoglobin (Haldane effect), metabolic shifts of pH, temperature and hemoglobin concentration. In addition, it is essential to consider possible sources of errors in the measurement of PCO_2_, including contamination of the samples with fluid or air bubbles, and insufficient precision of the gas analyzer. When comparing successive determinations of Pv-aCO_2_ gap, it is therefore recommended to consider only variations of at least ± 2 mmHg as real changes [43].

Two additional confounders in the interpretation of the Pv-aCO_2_ gap require some discussion. The first is hyperoxia. It has been observed that, in patients with circulatory shock, ventilation at 100% inspired oxygen fraction (FiO_2_) for 5 min increased venous PCO_2_, and hence the Pv-aCO_2_ gap, independent of changes in the hemodynamic status [44]. While this observation may be explained by a lower CO_2_ affinity of hemoglobin due to elevated venous PO_2_ (Haldane effect) [44], it may also reflect some impairment in microcirculatory blood flow, owing to the vasoconstrictive effects of hyperoxia [45]. The second confounder is acute hyperventilation with respiratory alkalosis. For example, as shown by Mallat et al. in 18 stable septic shock patients [46], an acute decrease in arterial PCO_2_ from 44 to 34 mmHg produced by transient hyperventilation (30 min) induced a significant increase in PCO_2_ gap (absolute 2.2 mmHg, relative + 48.5%). Possible mechanisms include, first, increased aerobic production of CO_2_ due to stimulated aerobic glycolysis under conditions of cellular alkalosis, and second, a reduction in microcirculatory blood flow due to the acute drop of CO_2_. Thus, both acute hyperoxia and hypocapnia may be important confounders in the interpretation of an increased Pv-aCO_2_ gap, which must be taken into account by the clinician.

## Conclusion

**Fig. 4:**
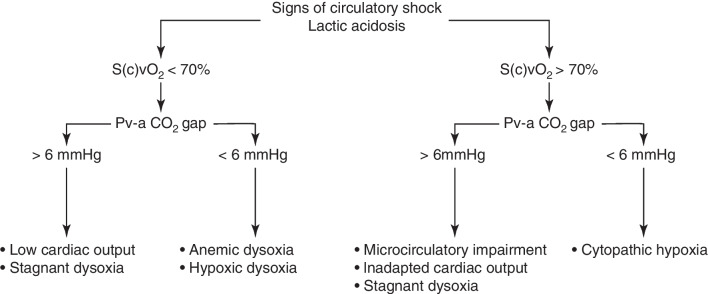
Usefulness of the Pva-CO_2_ gradient under conditions of circulatory shock. Proposed diagnostic algorithm integrating lactate, mixed (central) venous oxygen saturation (S(c)vO_2_) and the Pva-CO_2_ gap in patients with circulatory shock

**Fig. 5:**
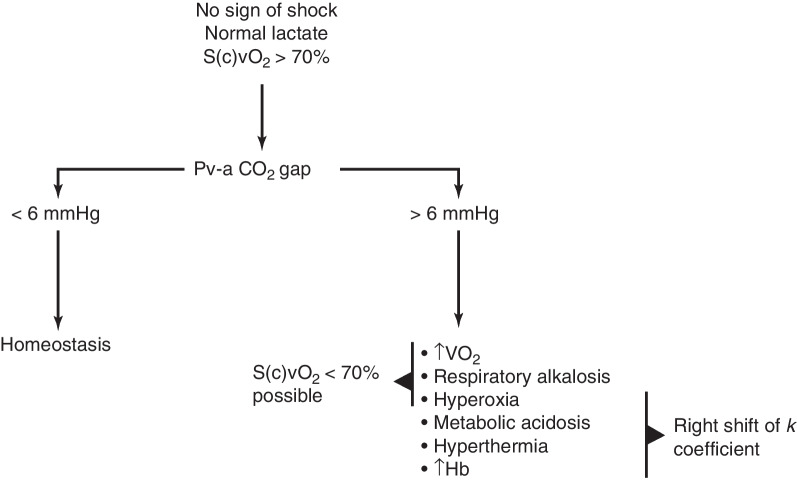
The Pva-CO_2_ gradient in the absence of circulatory shock. Proposed diagnostic algorithm to interpret an elevation in the Pva-CO_2_ gap in the absence of circulatory shock and with normal blood lactate. *S(c)vO*_*2*_ mixed (central) venous oxygen saturation

The Pv-aCO_2_ gap is a reliable indicator of impaired tissue perfusion, whether the result of a global reduction in cardiac output or to microcirculatory abnormalities, but it does not track tissue dysoxia, unless related to a stagnant mechanism. Being easily accessible and readily available, the Pva-CO_2_ gap should be included in the integrated evaluation of the patient in circulatory shock. Several diagnostic algorithms incorporating Pva-CO_2_ gradients have been proposed, such as those presented in Figs. 4 and 5. It remains to be established whether the Pva-CO_2_ gap should be part of a resuscitation bundle protocol, and whether therapies aimed at normalizing an increased Pva-CO_2_ gap could improve the dismal prognosis of circulatory shock.

## Data Availability

Not applicable.
